# A novel software for method comparison: MCS (method comparison software)—assessing agreement between estimated fetal weights calculated by Hadlock I–V formulas and birth weight

**DOI:** 10.1007/s00404-024-07680-2

**Published:** 2024-08-30

**Authors:** Şeyma Yaşar, Ahmet Kadir Arslan, Büşra Berfin Polat, Rauf Melekoğlu, Cemil Çolak, Saim Yoloğlu

**Affiliations:** 1 Medicine Faculty, Department of Biostatistics and Medical Informatics, Inonu University, Malatya, Türkiye; 2 Faculty of Medicine, Department of Obstetrics and Gynecology, Inonu University, Malatya, Türkiye

**Keywords:** Bland–Altman, Scaled summary indices, Regression methods, Hadlock formulas, Birtweight

## Abstract

**Introduction:**

The evaluation of the performance of new methods, expected to provide cheaper and faster results than existing (reference) methods in the health field, is based on comparing the results obtained with this new method to those obtained with the existing method. The primary aim of this study is to examine the correlational and absolute agreement between measurement methods in clinical studies using Bland–Altman analysis and methodological (Ordinary Least Squares, Weighted Ordinary Least Squares, Deming, Weighted Deming, Passing–Bablok, Theil-Sen, and Passing–Bablok for Large Data Sets.) methods, and the secondary aim is to compare the accuracy and precision of Hadlock (I–V) formulas used for fetal weight estimation.

**Materials and methods:**

The study was conducted on singleton pregnancies examined in the Prenatal Diagnosis and Treatment Unit of the Department of Obstetrics and Gynecology at Inonu University Faculty of Medicine and who gave birth in the Obstetrics Unit between 01.01.2020 and 01.09.2023, whose gestational ages were confirmed by first-trimester ultrasonography. Estimated fetal weights were calculated using Hadlock (I–V) formulas, and the agreement of these weights with birth weight was evaluated with Bland–Altman method.

**Results:**

The comparison of estimated fetal weights calculated using Hadlock formulas with birth weight was analyzed using Bland–Altman analysis, ICC, and CCC values along with regression analyses. According to the mean difference values obtained by Bland–Altman analysis, the estimated fetal birth weights obtained by the Hadlock IV formula were most consistent with the actual birth weights.

**Conclusions:**

The estimated fetal weights obtained using the Hadlock IV formula resulted in the closest measurements to the birth weight. This study showcases the efficacy of a new web-based software, Method Comparison Software (MCS), which can be utilized for evaluating the agreement between different methods in clinical measurements.

**Supplementary Information:**

The online version contains supplementary material available at 10.1007/s00404-024-07680-2.

## What does this study add to the clinical work

**Table:** 

Among the five different Hadlock formulas evaluated with the free web-based MCS statistical tool developed to evaluate the compatibility between new and reference methodologies in health research, the measurements obtained with the Hadlock IV formula gave the closest results to birth weight, thus supporting the use of this formula for accurate prenatal care and outcomes.	

## Introduction

The performance of new health methods, which aim to provide cheaper and faster results through technological advancements, is assessed by comparing their accuracy and precision to existing methods. Method comparison studies evaluate the fit between new and current methods. Complete agreement between different measurement methods is rare, but it is possible to understand how the new method differs from the reference method. If the differences do not affect clinical interpretation, the new method can replace or complement the standard method. Differences may arise from random or systematic errors. Traditional statistical approaches like correlation analysis, significance tests, and regression analysis are commonly used to evaluate the agreement between measurements from different methods [1, 2]. The correlation analysis shows the linear relationship between two measurements but cannot determine bias. Similarly, hypothesis testing accounts for constant error but does not address proportional error [3, 4]. The purpose of regression analysis is to infer the relationship between the independent variable *X* and the dependent variable *Y* [5]. In method comparison studies, it is unclear which measurement is dependent or independent. If regression is used, it assumes errors are only in the *Y* (dependent) method, not the *X* (independent) method, leading to inaccuracies in assessing method agreement [6]. Considering the studies and the results obtained, it has been observed that these two approaches give erroneous results in evaluating the agreement between the two measurements and alternative methods have been proposed. Bland–Altman analysis and methodological methods (Ordinary Least Squares, Weighted Ordinary Least Squares, Deming, Weighted Deming, Passing–Bablok, Theil-Sen and Passing–Bablok for Large Data Sets) are alternative to classical methods that are frequently used in method comparison studies. They allow for the examination of the relational agreement as well as the absolute agreement between the methods being compared, thereby enabling an understanding of the analytical methods and estimating the possible systematic error between them. Among these methods, Bland–Altman analysis evaluates the absolute fit between methods, while regression methods evaluate the relational fit between methods. Bland–Altman analysis and regression methods used to evaluate the agreement between methods differ depending on the purpose for which the agreement between measurements is evaluated. Regression analyzes can be used to adjustment/calibrate results from one measurement method against another, while the Bland–Altman method can be used to determine whether one method can be used safely in place of another, especially in clinical practice [7].

Perodic monitoring of fetal growth is of great importance for a healthy pregnancy and birth process [8]. Therefore, pregnant women should have regular prenatal assessments and ultrasound evaluations as recommended by their clinicians. These evaluations provide crucial information on fetal development, detect early problems, and guide appropriate interventions. This process helps protect the health of both mother and baby by identifying abnormal fetal growth and avoiding unnecessary, risky interventions. Failure to achieve normal fetal growth increases the risks of stillbirth, birth complications, metabolic issues, and long-term developmental problems [9, 10]. The formulations used for fetal weight estimation are also a part of this process, and their compatibility with the actual birth weight is of vital importance in accurately evaluating the process in question. There are many formulas developed for fetal weight estimation, such as the Hadlock (I–V) formulas, which are based on fetal biometry measurements [11–13].

The aim of this study is to develop a new user-friendly web-based software that allows the relational and absolute agreement between measurement methods in clinical research to be examined by Bland Altman analysis, Scaled Summary Indices (Intraclass Correlation Coefficient and Concordance Correlation Coefficient) and methodological methods (Ordinary least squares, Weighted least squares, Deming, Weighted Deming, Theil-Sen, Passing Bablok, and Passing–Bablok regression methods for large datasets) and the secondary aim is to compare the accuracy and precision of Hadlock (I–V) formulas used for fetal weight estimation.

## Materials and methods

### Dataset

The data set used in this study consists of all women with singleton pregnancies who were examined at the Prenatal Diagnosis and Treatment Unit of the Department of Obstetrics and Gynecology, Faculty of Medicine, Inonu University, who gave birth in the same department between 01/01/2020 and 01/09/2023 and whose gestational age was confirmed by first trimester ultrasonography. Women with multiple pregnancies, fetal death, major fetal anomalies (anomalies that were fatal or required prenatal or postnatal surgery), chromosomal abnormalities, genetic syndromes, macroscopic placental abnormalities, fetuses with ascites, skin edema or hydrops fetalis, premature rupture of membranes and amniotic fluid abnormalities were excluded from the study. The ultrasonographic measurements of the fetus and maternal measurements were performed within 72 h before delivery by a single clinician experienced in fetal ultrasonography screening. Measurements were made using a GE Voluson E6 (GE Medical Systems, Milwaukee, WI, USA) ultrasonography device and RAB2-5L (2–5 MHz) probe. The measurements were carried out in accordance with the methods defined in the “ISUOG Practice Guidelines: performance of the routine mid-trimester fetal ultrasound scan” guide published by the International Society of Ultrasound in Obstetrics and Gynecology (ISUOG) in 2011 and 2022 [14]. Biparietal diameter (BPD), head circumference (HC), abdominal circumference (AC) and femur length (FL) are biometric measurements used in Hadlock (I–V) formulations. For birthweight measurement, the baby was weighed on an electronic scale while completely naked. The Hadlock (I–V) formulas used to estimate fetal weight based on ultrasonic fetal biometry in this study are given in Table 1. The Inonu University Health Sciences Non-Interventional Clinical Research Ethics Committee approved this study (approval number: 2024/5773).

**Table 1:** Formulas for calculation of estimated fetal weight

Authors	Parameters	Formula
HadlockI [15]	BPD, HC, AC, FL	${\text{log}}_{10}\text{EFW}=1.3596 + 0.0064\text{xHC }+ 0.0424\text{xAC}+0.174\text{xFL}+0.00061\text{x BPDxAC}-0.00386\text{xACxFL }(\text{g},\text{cm})$
HadlockII [15]	AC, FL	${\text{log}}_{10}\text{EFW}=1.304+0.05281\text{xAC}+0.1938\text{xFL}-0.004\text{xACxFL }(\text{g},\text{cm})$
HadlockIII [15]	BPD, AC, FL	${\text{log}}_{10}\text{EFW}=1.335-0.0034\text{xACxFL}+0.0316\text{xBPD}+0.0457\text{xAC}+0.1623\text{xFL }(\text{g},\text{cm})$
HadlockIV [15]	HC, AC, FL	${\text{log}}_{10}\text{EFW}=1.326-0.00326\text{xACxFL}+0.0107\text{xHC}+0.0438\text{xAC}+0.158\text{xFL }(\text{g},\text{cm})$
HadlockV [16]	BPD, AC	${\text{log}}_{10}\text{EFW}=1.1134+0.05845\text{xAC}-0.000604{\text{xAC}}^{2}-0.007365{\text{xBPD}}^{2}+0.000595\text{xBPDxAC}+0.1694\text{xBPD }(\text{g},\text{cm})$

### The Bland–Altman method

The Bland–Altman method has been published by J Martin Bland and Douglas G. Altman as a series of studies evaluating the fit with simple calculations and graphical approaches. The approach in the Bland–Altman method is to graphically show the difference between the averages of the values obtained from the two measurement methods and the differences of these measurements [17]. If there is no relationship between the differences and the means, the fit between the two methods can be examined using the mean of the differences and standard deviation. The assumptions of the Bland–Altman method are that the difference measurements fulfill the assumptions of normal distribution and that there is no statistical relationship between the difference values and the mean values of the measurements. If two assumptions are provided, an examination of the fit between the two methods can be calculated using the mean and standard deviation values of the difference measures [18].

### The scaled summary indices

The reliability of a measurement method is defined as the high correlation between repeated measurements obtained with that method. Intra-class correlation coefficient or harmony relationship coefficient is used when investigating the harmony between more than one measurement obtained by a numerical measurement method or the harmony between different methods measuring the same value.

#### Intraclass correlation coefficient (ICC)

ICC was developed to determine the amount of correlation between measurements belonging to the same class (such as weights of children born in one litter, blood measurements for twins). ICC is a correlation coefficient obtained by using the variance estimates obtained by dividing the total variance into intra-group and inter-group variance. There are different ICC estimates depending on the study design (one-way random effects model, two-way random effects model, two-way mixed effects model), purpose (consistency, absolute agreement) and the way the measurements used were obtained (single measurement, total/average measurement) [19]. The one-way random effects model assumes that *k* measurements for each *n* subjects were made by *n* × *k* different observers and is more commonly used when the order of the measurements is not important. In the two-way random effect model, *n* number of subjects are evaluated by *k* number of observers randomly selected from a large population. In the two-way mixed effects model, where the observer effect is assumed to be constant, *n* number of subjects are evaluated by *k* number of observers. The conceptual difference between consistency and absolute agreement is explained by how systematic variability resulting from measurements or methods is evaluated. If the variability is insignificant, the variance term related to this variability is not included in the denominator of the ICC formula and the resulting ICC is considered a “consistency” measurement. If systematic variability between measurements or observers is significant, measurement/observer variability is included in the denominator of the ICC formula and the calculated ICC is considered “absolute agreement”. Finally, ICC calculations also vary depending on the number of measurements taken on a subject. If one measurement is taken from a subject with each method, the “single” option is used. If more than one measurement is taken from a subject with each method, the “average” option is used [20].

#### Concordance correlation coefficient (CCC)

Lin’s concordance correlation coefficient, unlike ICC, is a coefficient used to investigate the concordance between continuous measurements or two categorical measurements obtained by two different methods. CCC measures where each pair falls on the 45º line. Includes accuracy (*C*_b_) and precision (*ρ*_c_) measurements [21, 22].

### Regression analyses

The other method used in clinical studies where the compatibility between the two methods is evaluated is regression analyses. Regression methods can be used for various purposes. The first of these is to calibrate the newly developed method according to the reference method. In this case, one of the results obtained by the two methods is considered as the dependent variable and the other as the independent variable. In this case, it is accepted that the independent variable does not contain measurement error, but the existing error is caused by the dependent variable and is constant within the standard deviation range of this variable. In addition, the slope coefficient value obtained from the regression equation is tested against the zero value. Another method is to determine the error between two measurements. In this case, it may be possible that the measurement values obtained by both methods contain errors at the same time. Therefore, the regression methods developed for use in such studies are called “Type II Regression Methods” [5]. One of the important results of regression methods is constant variance. If the error term variance changes depending on the changes in the independent variable, there is varying variance. The regression methods to be used in both cases differ. Ordinary Least Squares, Weighted Ordinary Least Squares, Deming, Weighted Deming, Passing–Bablok, Theil-Sen, and Passing–Bablok regression methods for Large Data Sets are used to evaluate the fit between the two methods in the developed web-based software.

#### Ordinary least squares regression/weighted ordinary least squares regression

The ordinary least squares regression method is a regression method that aims to minimize the sum of squares of error. In other words, it is trying to estimate the regression line equation that minimizes the sum of the residual squares with the data obtained from the sample by using the Ordinary Least Squares technique. This method provides an accurate estimation of the regression coefficients if some assumptions such as normal distribution fitness, constant variance, not containing extreme values are provided. The OLS technique is a suitable technique to use if the assumption of constant variance is provided in the regression analysis. The weighted least squares (WOLS) technique was developed as an alternative to the OLS technique when the linear regression model has a problem of heteroscedasticity. In case of heteroscedasticity, OLS estimations are unbiased, but statistical hypothesis tests lose their validity because variance and covariance estimations are not efficient (with minimum variance) [23].

#### Deming regression/weighted deming regression

The Deming regression, which is a highly recommended technique in clinical studies in recent years, is also known as type II parametric regression technique and is similar to standardized principal component analysis. In his work in 1943, Deming suggested minimizing the function that will give the line equation that best fits the observation values if both variables have error measurements. In the Deming regression technique, the error sum of squares (SSE) to be minimized is calculated as seen in Eq. 1.

1 $${\text{SSE}} = \sum \left[ {\left( {X_{i} - \hat{X}_{i} } \right)^{2} + \lambda \left( {Y_{i} - \hat{Y}_{i} } \right)^{2} } \right]$$

Deming regression technique offers the most appropriate regression line by considering the error in both variables. In order to estimate the regression line with the Deming technique, the value of λ in Eq. 1 must be known. The λ value, which is the ratio of the variances of the *X* and *Y* measurements, enables the determination of the angle that will minimize the sum of squares of the deviations on the line. That is, it simultaneously minimizes the sum of the squares of the errors of the *X* and *Y* measurements. Deming regression is appropriate in the case of fixed systematic bias, i.e. the systematic differences are independent of the level of measurement. However, in the presence of proportional bias, the weighted form of Deming regression is performed. In the weighted Deming regression method, the sum of the squares of the weighted deviations at a certain angle to the line is made the smallest [24, 25].

#### Passing–Bablok regression/Passing–Bablok regression for large datasets

Similar to the Deming regression method, the Passing–Bablok method depend on the assumption that the results from both methods investigated may have a certain error. In this method, which does not require the parametric structure requirement, the calculations to be made are made according to the sequence numbers of the measurements obtained from the methods and it is accepted that the tested method and the reference method are independent of each other. This method stands out in some cases where it is not appropriate to use other linear regression models. There are no assumptions about sample and error dispersion in the use of the method. In this regression analysis performed on the rank scores of the observations, the slope of the regression line is calculated over the median of all possible regression slopes [26]. Based on this estimation principle, the biggest advantage of the method is that it is effective against extreme values. In the case of extreme values, weighting does not work, as in methods such as weighted least squares and weighted deming regression. The non-parametric approach underlying the Passing–Bablok method allows modeling the relationship between laboratory methods in the presence of extreme values. In the Passing–Bablok regression method, the relationship between the variables obtained by two methods measuring the same thing should be linear, high and positive. It is also used when there is no information about the distributions of the variables. The Passing–Bablok method, similar to the Deming Regression method, assumes that both laboratory methods are measurements with random error. However, it is a more flexible approach compared to the Deming Regression method, as it does not assume that the errors have constant variance [27].

#### Theil-Sen regression

The method proposed by Theil in 1950 is one of the most commonly used non-parametric regression methods along with Sen’s correction. In order to find the ${\widehat{\beta }}_{1}$ statistic, which is the estimator of the *β*_1_ parameter, when the sample units are considered in pairs, the slopes of all cases are calculated by Eq. 2.

2 $$S_{{{\text{ij}}}} = \frac{{y_{{\text{j}}} - y_{{\text{i}}} }}{{x_{{\text{j}}} - x_{{\text{i}}} }}, \quad({\text{j}} > {\text{i}})$$

The statistic ${\widehat{\beta }}_{1}$, which is the estimator of the *β*_1_ parameter, is calculated as the median of the slope values. Therefore, the ${\widehat{\beta }}_{1}$ statistic will be Med [*S*_ij_]. The constant term for the regression model is found by substituting the (*x*_i_,*y*_i_) or (*x*_j_,*y*_j_) points of the i. or j. variables that give the Med [*S*_ij_] value [28].

### The developed web-based software

The web-based software was designed using shiny version 1.0.5 [29] package on the basis of the *R* programming language [30] and can be freely accessed in both English and Turkish at http://biostatapps.inonu.edu.tr/MCS. In the new software, MKinfer [31] package was used for Bland–Altman analysis and the assumption of normal distribution of the difference of measurements, which is one of the assumptions of Bland–Altman statistical analysis, were evaluated with the appropriate statistical test (Shapiro–Wilk/Kolmogorov Smirnov), and the existence of the relationship between the differences and the averages was checked by Pearson/Spearman correlation analysis. One of the basic assumptions of linear regression is that the residuals are distributed with equal variance at each level of the predictor variable. However, when this assumption is violated, there is heteroscedasticity in the residuals and the results of the regression become unreliable. To determine whether heteroscedasticity exists in the MCS, the “bptest” function was used for the “Breusch-Pagan Test” in the lmtest [32] package. On the other hand, extreme and outlier values present in the data are usually values that are significantly different from other data or do not fit the general trend. These can have negative effects on statistical analysis and machine learning models. Identifying and processing extreme and outlier values is an important part of the data preprocessing stages. In MCS, Mahalanobis distance was used for multivariate outlier detection in the chemometrics [33] package for this case. For the regression methods performed in the software, the deming, pbreg, and theilsen functions in the deming [34] package and the mcreg function in the mcr [35] package were used where appropriate. Finally, the appropriate functions in the openxlsx [36] and rmarkdown[37] packages were used to print the analysis results. Comparison of the developed web-based software (MCS) with other software (IBM SPSS, NCSS, MedCalc, GraphPad Prism, Minitab, *R*) containing method comparison analysis methods is given in Table 2.

**Table 2:** Comparison of MCS with other software

	IBM SPSS	NCSS	MedCalc	GraphPad prism	Minitab	R	MCS
Bland–Altman	No^1^	Yes^2^	Yes	Yes^2^	No^3^	Yes^4^	Yes
ICC	Yes	No	Yes	No	No	Yes^4^	Yes
CCC	No^5^	Yes	Yes	No	No	Yes^4^	Yes
OLSR	Yes	Yes	Yes	Yes	Yes	Yes^4^	Yes
WOLSR	Yes	No	No	No	Yes	Yes^4^	Yes
DR	No	Yes	Yes	Yes	Yes	Yes^4^	Yes
WDR	No	No	No	No	Yes	Yes^4^	Yes
PBR	No	Yes	Yes	No	No	Yes^4^	Yes
Theil-Sen Regression	No	No	No	No	No	Yes^4^	Yes
PBR for large datasets	No	No	No	No	No	Yes^4^	Yes
Commercial	Yes	Yes	Yes	Yes	Yes	No	No

^1^The differences and means of the measurements can be calculated in the same software, and then graphs can be drawn manually by checking assumptions

^2^Assumptions need to be checked

^3^Requires an additional macro

^4^Requires moderate coding knowledge

^5^Can be done using Syntax

There are six different main modules in MCS: “Input”, “Data Loading”, “Bland Altman Analysis”, “Scaled Summary Indices”, “Regression Methods” and “Quotation”. Brief information about MCS is given in the “Introduction” module. In the “Data Loading” menu, a data file with.xls/.xlsx or.sav extension is loaded and the type and role of the variables are determined. In the “Bland–Altman Analysis” module, the compatibility between methods is evaluated with Bland–Altman analysis and its graph is drawn. In addition, comments on the results are presented to the researcher. The main menu of “Scaled Summary Indices” consists of two submodules: “Intra-Class Correlation Coefficient” and “Concordance Correlation Coefficient”. In these two submodules, the relevant coefficients are calculated and the results are interpreted. In the “Regression Methods” module, the harmony of measurements obtained with different methods is determined by changing methods such as Ordinary Least Squares Regression, Weighted Ordinary Least Squares Regression, Deming Regression, Weighted Deming Regression, Passing–Bablok Regression, Passing–Bablok regression for large datasets, Theil-Sen Regression. It is carried out by taking into account the variance and the existence of outliers/extreme values. The analysis results made in each module are available for download in.xlsx. In this study, the compatibility of estimated fetal birth weights calculated using Hadlock I–V formulas with birth weight was evaluated using all methods in MCS, and screenshots of each module are given in the Supplementary Fig. 1–5.

## Results

Demographic characteristics of the women included in the study are given in Table 3.

**Table 3:** The descriptive statistics on demographic characteristics of women included in the study

Variables	Descriptive statistics	
	Mean ± SD	Median (min–max)
Age at Birth (years)	31.397 ± 5.519	31 (16–46)
Current age (years)	34.427 ± 5.655	34 (18–52)
Weight (kg)	72.217 ± 10.992	70 (50–127)
Height (cm)	164.271 ± 5.105	165 (150–180)
Gravida	2.579 ± 1.42	2 (0–10)
Parite	1.254 ± 1.172	1 (0–9)
Abortus	0.331 ± 0.712	0 (0–7)
Alive	1.23 ± 1.143	1 (0–8)

*SD* standard deviation, *Min* minimum, *Max* maximum

According to the assumption of Bland–Altman analysis, the difference values of fetal birth weights calculated by Hadlock I, Hadlock II, Hadlock III, Hadlock IV, and Hadlock V formulas are normally distributed (respectively, *p* = 0.102, *p* = 0.200, *p* = 0.053, *p* = 0.101, *p* = 0.105). On the other hand, the relationship between the mean and difference values of fetal birth weights calculated with the five formulations, which is another assumption, was examined with Sperman correlation coefficient and no statistically significant relationship was found (respectively, *p* = 0.618, *p* = 0.073, *p* = 0.074, *p* = 0.647, *p* = 0.773). Therefore, the results of the Bland–Altman analysis of birthweight and fetal birthweight calculated according to five different formulas are given in Table 4 with the developed web-based software (MCS).

**Table 4:** Bland–Altman analysis results for birthweight and Hadlock I-V formulations

Fetal weight estimation formula	Statistics	Differences	%95 CI
Hadlock I	Mean	− 45.67	[− 62.53, − 28.82]
	Lower limit	− 571.38	[− 600.2, − 542.57]
	Upper limit	480.04	[451.22, 508.85]
Hadlock II	Mean	− 68.75	[− 86.5, − 50.99]
	Lower limit	− 622.55	[− 652.9, − 592.19]
	Upper limit	485.05	[454.7, 515.41]
Hadlock III	Mean	− 66.21	[− 83.27, − 49.15]
	Lower limit	− 598.18	[− 627.34, − 569.02]
	Upper limit	465.76	[436.6, 494.92]
Hadlock IV	Mean	− 35.11	[− 52.05, − 18.16]
	Lower limit	− 563.64	[− 592.62, − 534.67]
	Upper limit	493.43	[464.46, 522.41]
Hadlock V	Mean	− 83.57	[− 101.43, − 65.72]
	Lower limit	− 640.3	[− 670.81, − 609.78]
	Upper limit	473.15	[442.63, 503.67]

*CI* confidence interval

**Fig. 1:**
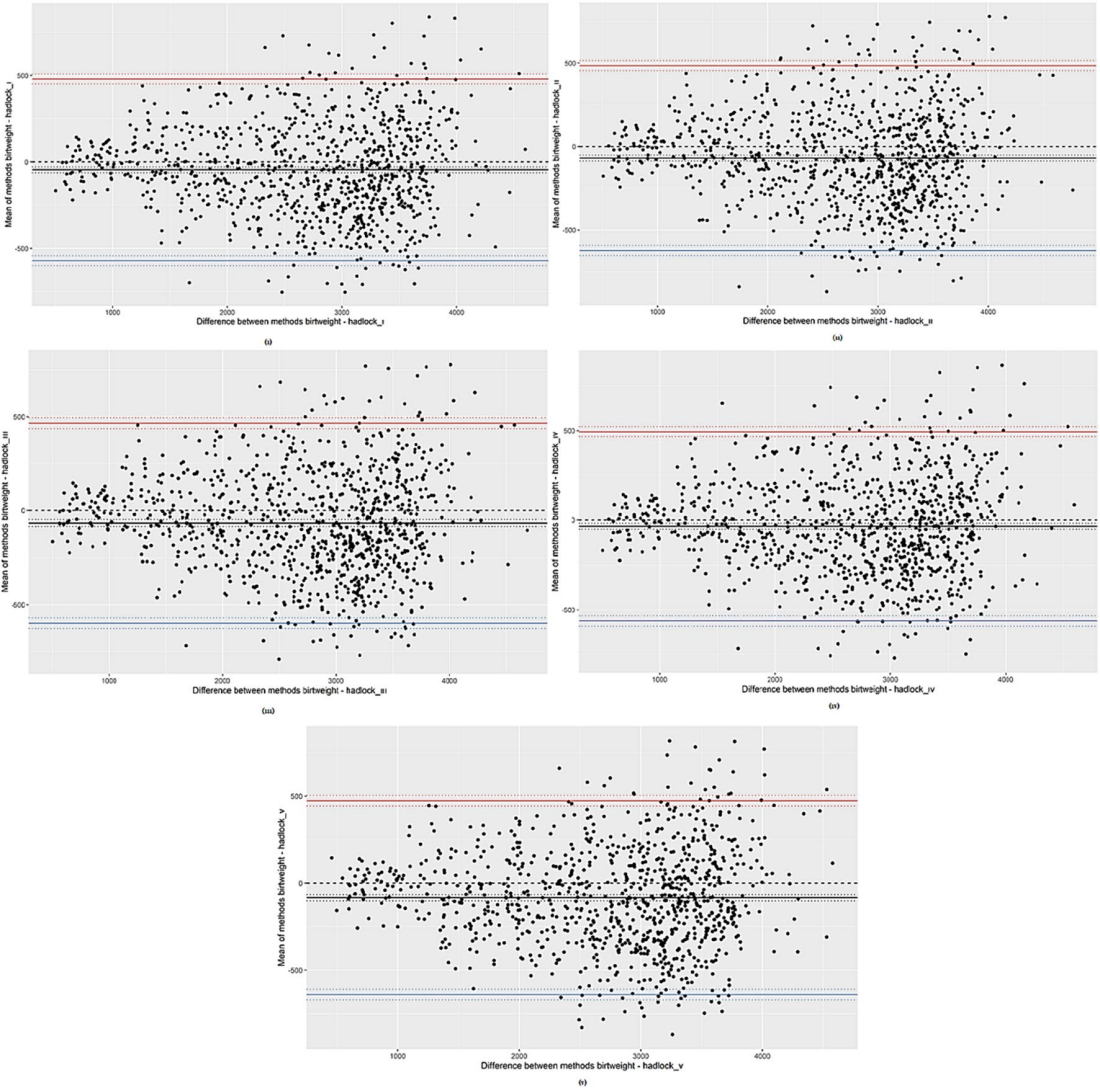
Bland–Altman plots of the agreement between estimated fetal weights and birth weight calculated by five different formulas based on biometric measurements; (**i**) Birthweight- Hadlock I; (**ii**) Birthweight- Hadlock II; (**iii**) Birthweight- Hadlock III; (**iv**) Birthweight- Hadlock IV; (**v**) Birthweight- Hadlock V

The mean differences estimate for Hadlock I is − 45.67, for Hadlock II it is − 68.75, for Hadlock III it is − 66.21, for Hadlock IV it is − 35.11 and finally for Hadlock V it is − 83.57. On the other hand, the limits of agreement at the 95% confidence level calculated by using the mean and standard deviation values of the difference between fetal birth weight and birth weight obtained with the formulas were estimated in the range of − 571.38–480.04 for Hadlock I, − 622.55–485.05 for Hadlock II, − 598.18–465.76 for Hadlock III, − 563.64–493.43 for Hadlock IV, and finally − 640.3–473.15 for Hadlock V. The estimated fetal weight obtained using the Hadlock I formula is 571.38 g more and 480.04 g less than the birth weight. The estimated fetal weight calculated using Hadlock II is 622.55 g more and 485.05 g less than the birth weight. Similarly, the estimated fetal weight calculated by Hadlock III is 598.18 g more and 465.76 g less than the birth weight. On the other hand, the estimated fetal weight calculated by Hadlock IV is 563.64 g more and 493.43 g less than the birth weight. Finally, the estimated fetal weight calculated by Hadlock V is 640.3 g more and 473.15 g less than the birth weight. Furthermore, Bland–Altman plots of the agreement between estimated fetal weights calculated by five different formulas based on biometric measurements and birth weight are given in Fig. 1.

In the measurements, it was assumed that the effect of the method was fixed and the subject effect was random, and the interaction between the method and the subject was ignored. In the datasets included in the study, since a single fetal weight was calculated with five formulas for each baby, single-measurement ICC values were taken into account. The results of the ICC values between the fetal weights calculated by the formula and birth weight are given in Table 5.

**Table 5:** The results of the ICC values between the fetal weights calculated by the formulas and birthweight

Formulas	ICC	95% CI		*F* value corresponding to test value = 0			*p*
		Lower level	Upper level	Value	*df1*	*df2*	
Hadlock I	0.95	0.94	0.96	40.79	974	578.67	** < 0.001**
Hadlock II	0.94	0.93	0.95	37.09	974	257.68	** < 0.001**
Hadlock III	0.95	0.94	0.96	40.28	974	254.91	** < 0.001**
Hadlock IV	0.95	0.94	0.96	40.03	974	794.83	** < 0.001**
Hadlock V	0.94	0.93	0.95	36.20	974	145.20	** < 0.001**

The bold symbol indicates that the ICC value is statistically significant

*ICC* interclass correlation coefficient, *CI* confidence interval

Considering the results in Table 5, there is excellent agreement between estimated fetal weights and birthweights obtained with Hadlock I, Hadlock III, and Hadlock IV formulas, while there is high agreement between estimated fetal weights and birthweights obtained with Hadlock II and Hadlock V formulas. On the other hand, the results of the CCC coefficients, in which the concepts of “accuracy” and “precision” were also evaluated to assess the agreement between these five different measurements and birth weight, are given in Table 6.

**Table 6:** The results of the CCC values between the fetal weights calculated by the formulas and birthweight

	CCC	95% CI		ρ* (accuracy)	*C*_b_** (precision)
		Lower limit	Upper limit		
Birthweight/Hadlock I	0.951	0.945	0.957	0.952	0.998
Birthweight/Hadlock II	0.945	0.938	0.951	0.948	0.996
Birthweight/Hadlock III	0.949	0.943	0.956	0.952	0.997
Birthweight/Hadlock IV	0.950	0.944	0.956	0.951	0.999
Birthweight/Hadlock V	0.942	0.935	0.949	0.946	0.995

*CCC* concordance correlation coefficent, *CI* confidence interval

*Pearson correlation coefficient

**Bias correction factor

There was high agreement between the estimated fetal weights calculated by Hadlock I, Hadlock III and Hadlock IV formulas and birth weights, while there was moderate agreement between the estimated fetal weights calculated by Hadlock II and Hadlock V formulas and birth weights (Table 6). In addition, the accuracy and precision values for the measurements obtained from all formulas were considerably higher. Before examining the agreement of estimated fetal weights calculated with five different formulations with birth weight using regression methods, variance was assessed using the Brush-Pagan test and variance was found to vary between all pairs of measurements (*p* < 0.001). On the other hand, Weighted Least Squares and Weighted Deming Regression methods based on multivariate outlier detection based on Mahalonobi distance are also not suitable for evaluating the agreement. Therefore, the fit between Hadlock I–V and birth weight will be evaluated by Passing–Bablok and Theil-Sen regression methods. The table of values for regression methods is given in Table 7.

**Table 7:** Passing Bablok and Theil-Sen regression methods results

Regression type	Formulas	Components of the equation	Bias	95% CI	Regression equations
Passing-Bablok regression	Hadlock I	Intercept-constant	44.269	21.647 to 63.094	$\widehat{y}$ = 44.269 + 1.006*x*
		Slope-proportional	1.006	0.998 to 1.014	
	Hadlock II	Intercept-constant	24.329	2.086 to 49.562	$\widehat{y}$ = 24.329 + 1.019*x*
		Slope-proportional	1.019	1.01 to 1.028	
	Hadlock III	Intercept-constant	36.538	15.362 to 57.192	$\widehat{y}$ = 36.538 + 1.019*x*
		Slope-proportional	1.019	1.011 to 1.028	
	Hadlock IV	Intercept-constant	51.288	26.942 to 74.766	$\widehat{y}$ = 51.288 + 0.997*x*
		Slope-proportional	0.997	0.989 to 1.006	
	Hadlock V	Intercept-constant	86.621	62.962 to 107.897	$\widehat{y}$ = 86.621 + 1.005*x*
		Slope-proportional	1.005	0.996 to 1.014	
Theil-sen regression	Hadlock I	Intercept-constant	176.274	123.851 to 225.369	$\widehat{y}$ = 176.274 + 0.953*x*
		Slope-proportional	0.953	0.933 to 0.973	
	Hadlock II	Intercept-constant	168.493	113.667 to 227.786	$\widehat{y}$ = 168.493 + 0.961*x*
		Slope-proportional	0.961	0.952 to 0.97	
	Hadlock III	Intercept-constant	168.057	114.073 to 220.383	$\widehat{y}$ = 168.057 + 0.965*x*
		Slope-proportional	0.965	0.956 to 0.973	
	Hadlock IV	Intercept-constant	181.344	133.51 to 234.811	$\widehat{y}$= 181.344 + 0.945*x*
		Slope-proportional	0.945	0.936 to 0.953	
	Hadlock V	Intercept-constant	243.371	176.4 to 296.891	$\widehat{y}$ = 243.371 + 0.944*x*
		Slope-proportional	0.944	0.935 to 0.954	

*x* Birtweight, *y* The measurement method in the row (Hadlock I, Hadlock II, Hadlock III, Hadlock IV, and Hadlock V)

**Fig. 2:**
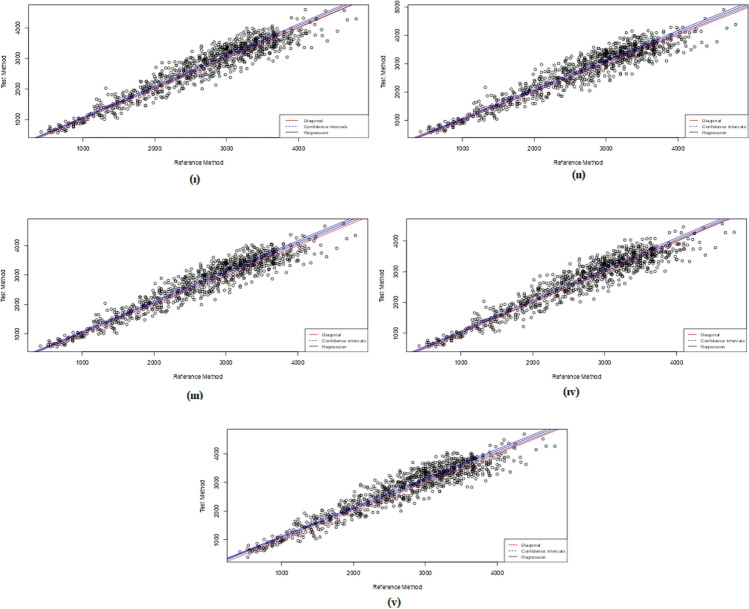
The Passing–Bablok regression plots for Hadlock I–V and birthweight

**Fig. 3:**
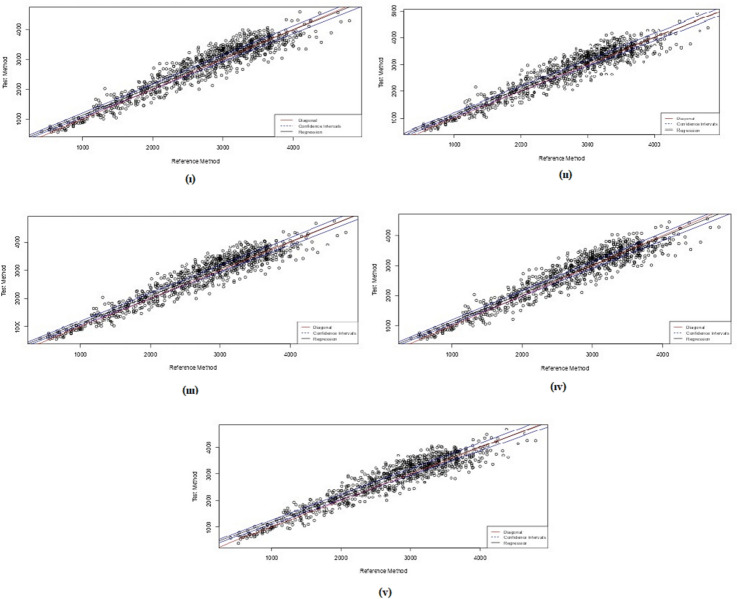
The Theil-Sen regression plots for Hadlock I–V and birthweight

When the data in Table 7 are examined, according to the results of the Passing Bablok regression analysis, the confidence intervals for the cut-off point of the regression coefficients examining Hadlock I, Hadlock II, Hadlock III, Hadlock IV and Hadlock V and birth weights do not include the value “one”. Therefore, systematic error is observed between the measurements. However, for Hadlock II and Hadlock III with Passing Bablok, the confidence intervals for the slope from the regression coefficients do not include the value "zero". Therefore, there is a proportional error between these two measurement methods and birthweight. The plots for Passing Bablok and Theil Sen regression are given in Figs. 2 and 3, respectively.

## Discussion

The comparison of a newly developed method in the field of health with reference methods is an important step in assessing the reliability and effectiveness of the method. In this field, there are many software (IBM SPSS [38], NCSS (https://www.ncss.com/software/ncss/), MedCalc [39], GraphPad Prism [40], Minitab [41], R [30], etc.), most of them commercial, which include some of the methods used to determine the statistical agreement between methods and also developed for statistical methods used in data analysis. The Bland Altman analysis is one of the standard procedures available in most statistical analysis software such as IBM SPSS, NCSS, MedCalc, UNISTAT Statistics [42], GraphPad Prism, Minitab and R, and offers different features for Bland–Altman analysis. IBM SPSS, the most widely used commercial software, has not developed a special module for Bland–Altman. The differences and means of the measurements for which agreement is being investigated can be calculated in the same software, and then graphs can be drawn manually by checking assumptions. NCSS provides several calculations for Bland–Altman analysis, including the Bland–Altman graph for the compared methods and confidence intervals for the difference values. However, like SPSS, all three software programs require assumptions to be checked individually. On the other hand, another commercial software, MedCalc, in addition to the features in these three software, uses a paired sample t-test to test for the presence of a consistent deviation between two measurements. Similarly, the commercial Minitab software does not have a module in itself, but performs the Bland–Altman analysis with an additional downloaded macro. Finally, R, an open source, free programming language, requires moderate coding knowledge to perform Bland–Altman analyses. On the other hand, Bland–Altman analyses can also be performed with a web-based tool called BA-plotteR and a paid statistical analysis add-in for Microsoft Excel called Analyze-it (https://analyse-it.com/). However, these tools do not state researchers how to interpret the results. The new software, MCS, differs from other existing software in that it is free of charge, can test the assumptions of Bland–Altman analysis, plot graphs, and interpret the results in a way that researchers can better understand.

Similarly, while the ICC coefficient, which evaluates the agreement between measurements, can be calculated using IBM SPSS, MedCalc software and *R* programming languages, it cannot be calculated in NCSS, GraphPad Prism and Minitab software. On the other hand, while another coefficient, the CCC coefficient, can be calculated using NCSS, MedCalc software and the *R* programming language, it cannot be calculated in IBM SPSS, GraphPad Prism and Minitab software. However, for calculations made in the *R* programming language, intermediate level coding knowledge is required. In the developed web-based software (MCS), calculation of both coefficients and interpretation of the obtained value are available. Finally, while the OLSR and WOLSR methods are available in IBM SPSS software, there is no module for the DR, WDR, PBR, Theil-Sen and PBR for large datasets methods. On the other hand, while there are OLSR, DR and PBR methods in the NCSS and MedCalc softwares, the weighted version of these methods (i.e. WOLSR, WDR) and Theil-Sen, PBR for large datasets methods are not available. As GraphPad Prism has a menu for OLSR and DR methods, WOLSR, WDR, which are the weighted versions of these two methods, and PBR, Theil-Sen, PBR for large datasets analyzes cannot be performed. Finally, while OLSR, WOLSR, DR and WDR methods are available in Minitab software for the harmony between values obtained with different methods, PBR, Theil-Sen Regression, PBR for large datasets methods are not available. All of these methods can be implemented in *R*, a free programming language. However, in order to apply these methods, intermediate level coding knowledge is required. The new web-based software developed includes all these regression methods and provides information about systematic and proportional error according to the confidence intervals of the coefficients to the researcher in an explanatory manner. In addition, the presence of tests to determine the presence of heteroscedasticity and extreme/outlier values, which is a critical point in choosing the right regression methods to evaluate the fit between methods, stands out as a significant difference of the developed software from other tools. Finally, MCS is a free software that does not require any registration or subscription, does not require coding knowledge, is web-based, and can be used by researchers, regardless of the operating system, to examine the compatibility of the results obtained with different methods, especially in clinical measurements.

Since this study examines the compatibility of estimated fetal weights obtained with five different formulas (Hadlock I–V) with birthweight, the results of Bland–Altman analysis will be taken into account. According to the results of the Bland–Altman analysis obtained with MCS, the measurements obtained with the Hadlock IV formula, which had the lowest mean difference value, obtained the closest measurements to the birthweight. In the light of the findings, the estimated fetal weight calculated by Hadlock IV may be 563.64 g more and 493.43 g less than the birthweight. On the other hand, according to the ICC and CCC values between Hadlock IV and birth weight, it can be said that there is almost perfect agreement between the two measurement methods.

The determination of fetal weight through sonographic means is a significant aspect within the field of obstetrics. The significance of utilizing sonograms for predicting fetal weight becomes more pronounced starting from the 24th week of gestation, also known as the gestational age of viability. Estimated fetal weight (EFW) stands as a crucial parameter aiding decision-making processes during the perinatal period, and it can be computed through various mathematical approaches [11, 43]. The initial weight estimation formulae were initially reliant on the measurement of the biparietal diameter (BPD). Subsequent progress in deriving various mathematical formulas incorporating multiple biometric indicators such as biparietal diameter (BPD), head circumference (HC), abdominal circumference (AC), and/or femur length (FL) provided a significant improvement to estimate fetal weight compared to traditional clinical estimation techniques like inspection and palpation [44]. The vast majority of weight estimation formulas are typically derived through regression analyses conducted on relatively small cohorts of average term infants. The average term infants play a crucial role in the development of these formulas. The magnitude of the mean percentage error (MPE) and mean absolute percentage error (MAPE) in the formulas is closely linked to the weight of the infants. It is not unexpected to observe a clinically significant level of error susceptibility in weight estimations for both underweight infants, pre-term infants, and infants with macrosomia. The precision of these formulas may differ, and incorrect values could impact medical practitioners’management. The efficacy of these formulas might also fluctuate for macrosomic or low birth weight fetuses [45, 46]. The presence of intrauterine growth restriction and macrosomia presents significant hazards for the health outcomes of both the newborn and the mother. Understanding the anticipated fetal weight plays a crucial role in guiding the proper approach to managing these complications in obstetrics. Hence, utilizing the most dependable and precise formula is crucial for accurately predicting the fetal weight. Plonka et al. conducted a retrospective cohort study to assess the accuracy of 11 fetal weight prediction formulas, including the Hadlock I–V formulas. The authors utilized the R-Spearman correlation method to measure the strength of the relationship between the actual birth weight and the estimated fetal weight. Additionally, precision was determined through the application of the “limits of agreement” technique established by Bland and Altman. Among fetuses weighing less than 2500 g, the Hadlock3 formula demonstrated the highest precision with a Mean Absolute Percentage Error (MAPE) of 7.04%. Furthermore, Hadlock III displayed the greatest Spearman correlation coefficient (*R* = 0.845). The other Hadlock formulas also exhibited substantial accuracy, underscoring their reliability [47]. Hoopman and colleagues conducted a study to evaluate the precision and distribution of errors in 35 weight estimation equations designed for infants weighing between 2500 and 4000 g. Their aim was to determine which formulae exhibited the closest adherence to the demands of clinical practice. The researchers assessed the performance of these 35 established weight estimation formulae using a sample of 3416 fetuses falling within the specified weight range. The analysis involved comparing the average percentage error, absolute percentage error, and the proportion of estimates falling within error margins of 5, 10, 20, and 30%. The results indicated that the formulas yielding the lowest average percentage errors were the Hadlock III and V (0.8% and − 0.8%, respectively), with corresponding standard deviations of 9.2%–10.0% [48]. Esinler et al. compiled and analyzed the performance of 18 diverse fetal weight prediction formulas. They compared these formulas across the entire study cohort and various subgroups employing metrics like percentage error (PE), absolute percentage error (APE), and Cronbach’s alpha. The results of the study brought attention to the three smallest average APE values associated with Hadlock4 (9.1%), Hadlock1 (9.2%), and Ott (9.8%). The researchers reached a conclusion that the formulas Hadlock I, Hadlock III, and Ott demonstrated the most accurate predictions of estimated fetal weight (EFW) across all fetuses included in their investigation [49]. In contrast in this study, we demonstrated that within the various Hadlock formulas examined, the measurements derived from the employment of the Hadlock IV formula exhibited the least average disparity value and achieved the measurements most proximate to the actual birthweight. This discrepancy may be attributed to several key factors, including differences in the study populations and measurement techniques. Notably, our study cohort included a diverse range of maternal and fetal characteristics, potentially rendering the comprehensive parameters of the Hadlock IV formula more reflective of this variability. Moreover, the precision in ultrasound measurement techniques and the timing of assessments in our study may have further favored the accuracy of the Hadlock IV formula. It is also important to consider the statistical methods employed; our analysis meticulously accounted for outliers and variability, ensuring a robust comparison between predicted and actual birth weights. While the Hadlock III formula has demonstrated commendable performance across unselected and high-risk populations, its widespread adoption may also reflect historical precedence and the practicality of its use in clinical settings. However, the findings from our study suggest that when feasible, incorporating the Hadlock IV formula could enhance the precision of birth weight predictions, thereby improving prenatal care and outcomes. Acknowledging these factors is crucial in understanding the nuances behind the formula selection for fetal weight estimation and underscores the necessity for ongoing research to refine these predictive tools. Also, numerous opportunities exist for enhancing the accuracy of weight estimations. In the realm of sonographic measurements’ precision, multiple research endeavors have indicated that practical experience in sonography or targeted training on patients or simulators may lead to a reduction in errors. Additionally, a specific time frame between measurements and delivery, not exceeding seven days, has the potential to diminish errors. An alternative approach, particularly beneficial for heavier infants, involves refining weight estimations by amalgamating ultrasound findings with maternal and pregnancy-specific variables such as gestational age, parity, fetal gender, and maternal height or weight. Nevertheless, the perceived advantages of such a meticulous process are a topic of contentious debate within current scholarly discourse. Despite this, a consensus exists on the fact that maternal obesity escalates the margin of error in estimations.

## Limitations

This study has some methodological limitations that should be taken into account when interpreting the results and evaluating their generalizability. The first limitation of our study is that the pregnant women included in the study were not grouped according to race, age, weight, height, and presence of any comorbidities such as diabetes and blood pressure (before or during pregnancy), pregnancy week. In future studies, these groups can be taken into account during data collection and the agreement of both the aforementioned formulas (Hadlock I–V) and different estimated fetal weight calculation formulas with birth weight can be performed using MCS software.

## Conclusion

In conclusion, this study underscores the paramount importance of employing robust and accessible statistical tools like MCS for evaluating the concordance between novel and reference methodologies in healthcare research. The use of the developed free web-based MCS in method comparison studies will significantly improve the accuracy of the analyses performed and the efficiency of the results, and can be a comprehensive analytical tool suite that exceeds the capabilities of many commercial software packages. On the other hand, the results of the analysis obtained using the MCS software show that the measurements obtained with the Hadlock IV formula are closest to the birth weight, which supports the use of this formula for accurate prenatal care and outcomes.

## Supplementary Information

Below is the link to the electronic supplementary material.

Supplementary file1 (DOCX 1784 KB)

## Data Availability

The data that support the findings of this study are available from the corresponding author upon reasonable request.
