# Supplementary material for: A novel software for method comparison: MCS (method comparison software)—assessing agreement between estimated fetal weights calculated by Hadlock I–V formulas and birth weight
Source: Arch Gynecol Obstet. 2024 Aug 30;310(5):2439–52. doi: 10.1007/s00404-024-07680-2 (PMC11485033; doi:10.1007/s00404-024-07680-2)
Supplement: Supplementary file 1 — Supplementary file1 (DOCX 1784 KB) [file 404_2024_7680_MOESM1_ESM.docx]

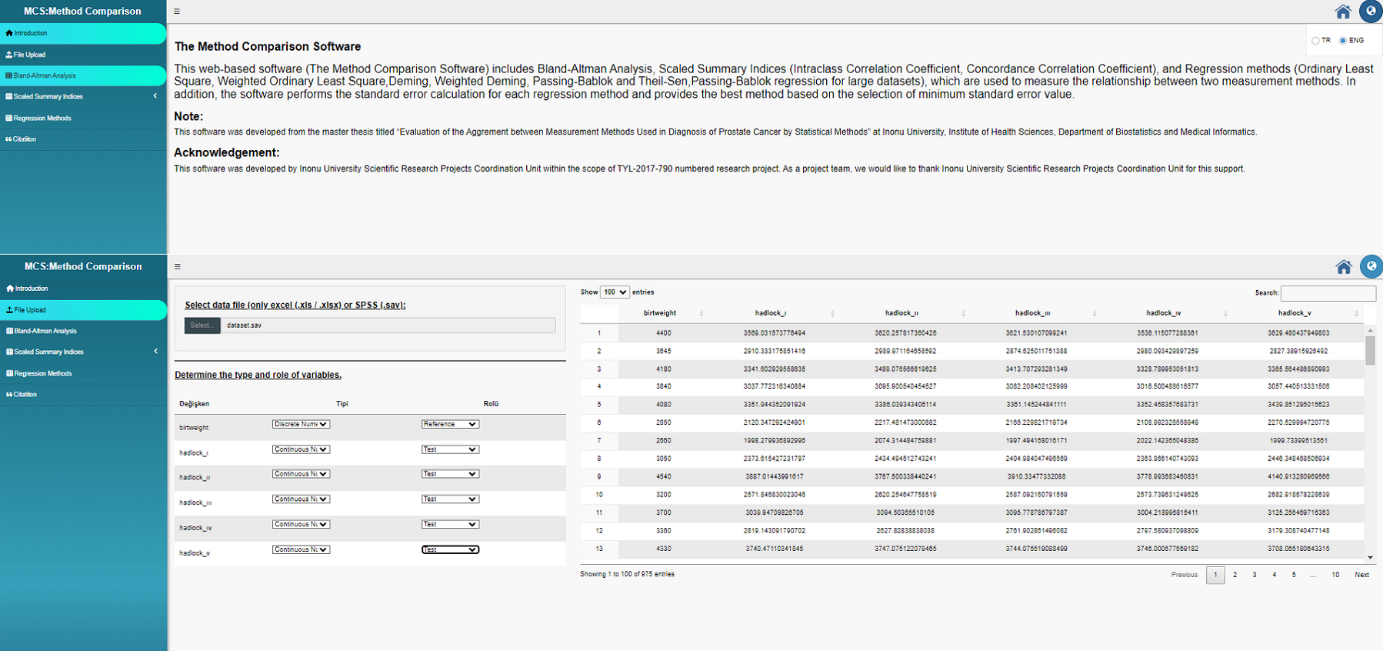


**Figure 1.** The screenshot of the "Introduction" and "File Upload" modules of the developed web-based software


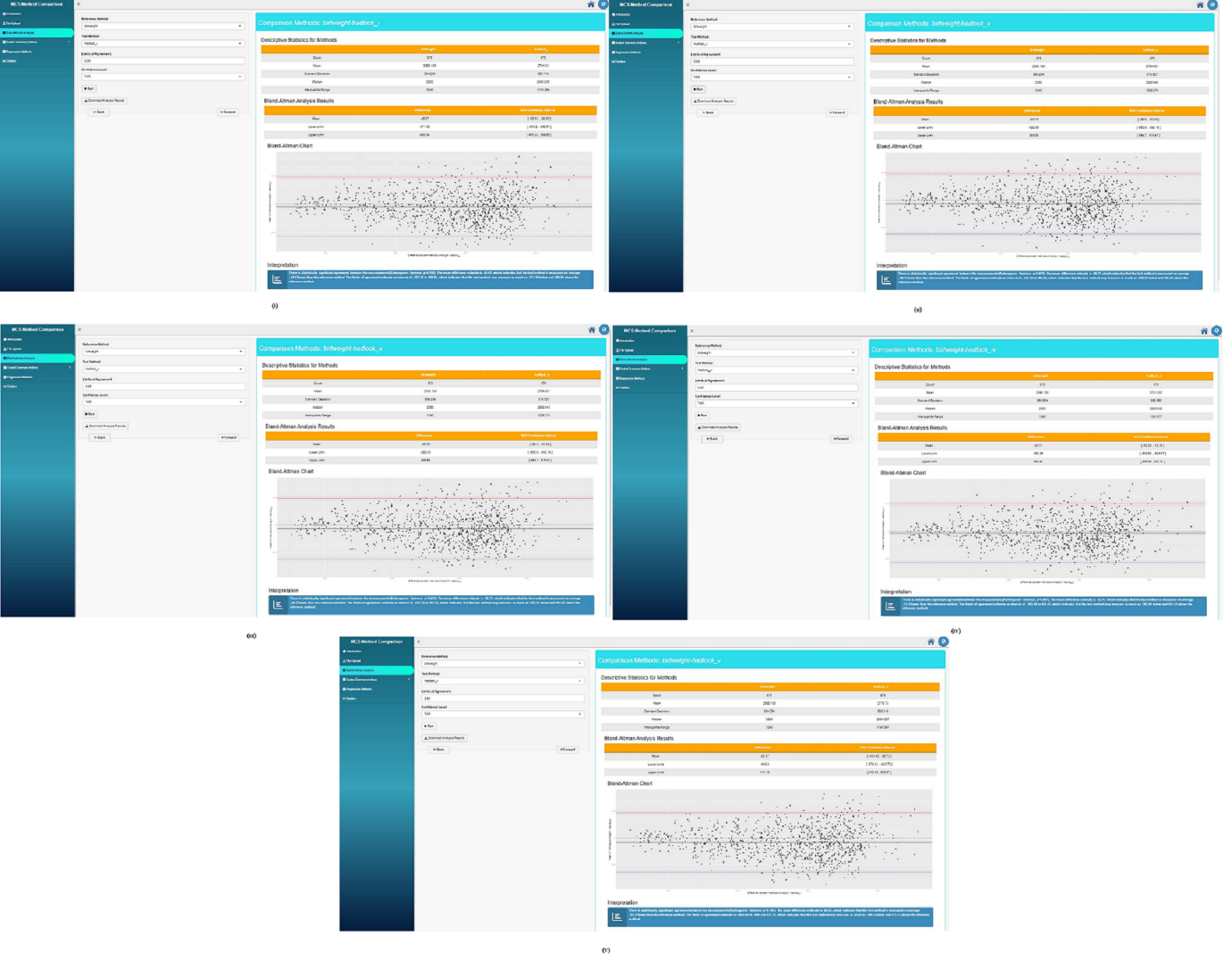


**Figure 2.** The screenshot of the "Bland Altman Analysis" module of the developed web-based software; (ı) Birthweight-Hadlock I; (ıı) Birthweight-Hadlock II; (ııı) Birthweight-Hadlock III; (ıv) Birthweight-Hadlock IV; (v) Birthweight-Hadlock V.

**
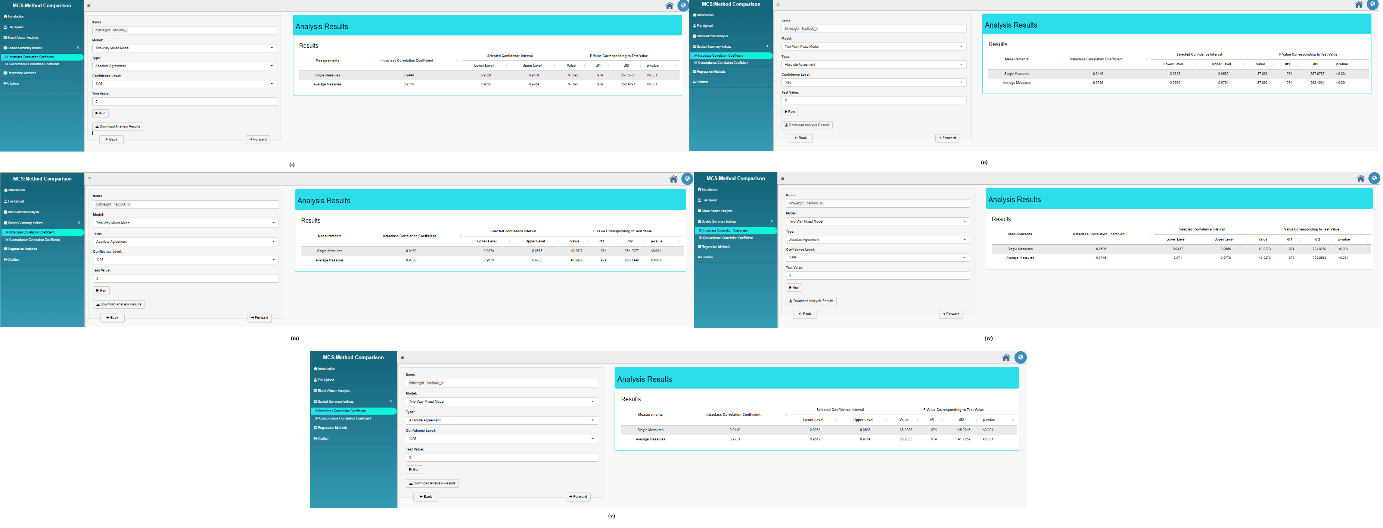
**

**Figure 3.** Screenshot of the "Interclass Correlation Coefficient" sub-module of the "Scaled Summary Indices" module of the developed web-based software; (ı) Birthweight-Hadlock I; (ıı) Birthweight-Hadlock II; (ııı) Birthweight-Hadlock III; (ıv) Birthweight-Hadlock IV; (v) Birthweight-Hadlock V.

**
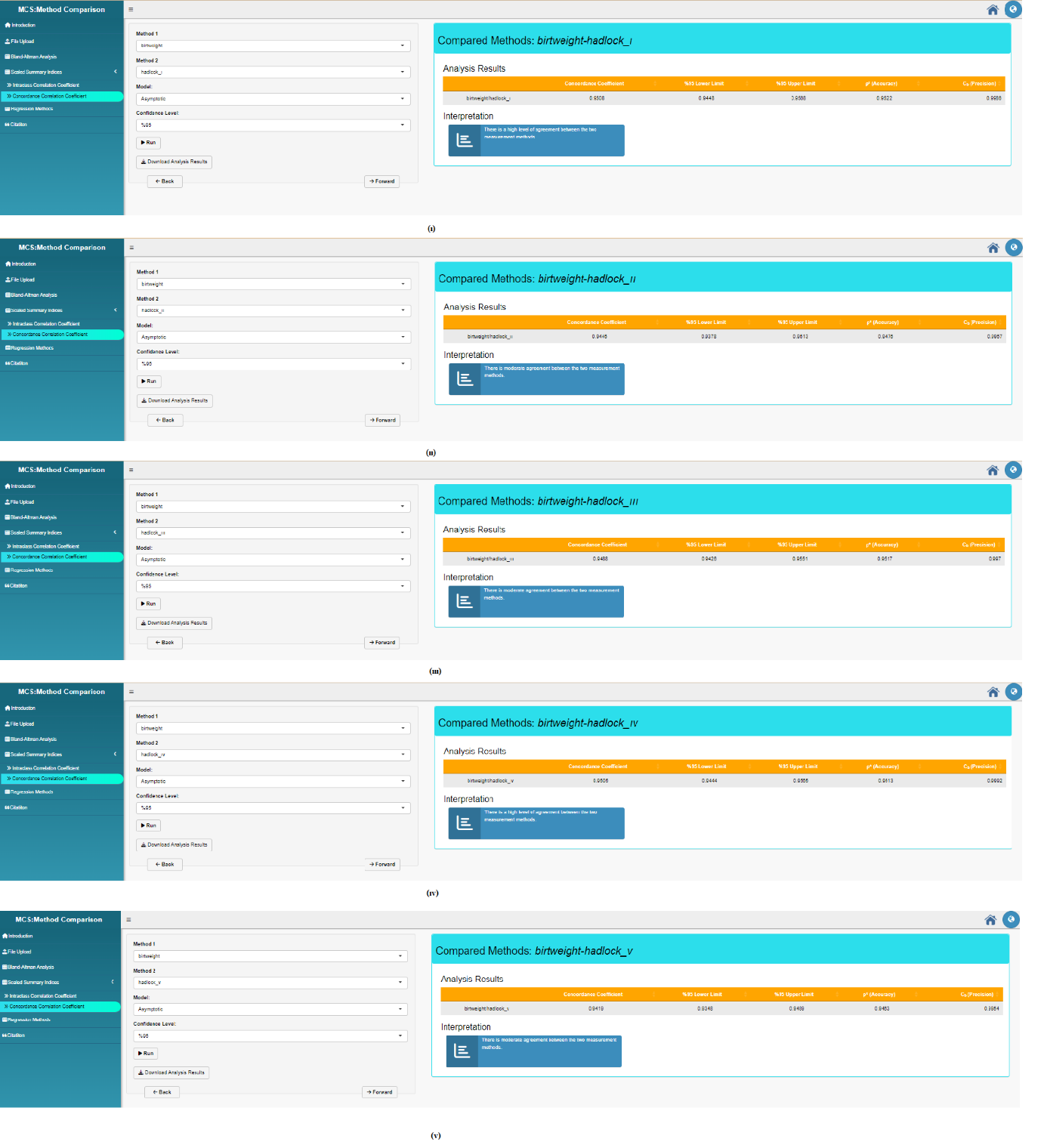
**

**Figure 4.** Screenshot of the "Concordance Correlation Coefficient" sub-module of the "Scaled Summary Indices" module of the developed web-based software; (ı) Birthweight-Hadlock I; (ıı) Birthweight-Hadlock II; (ııı) Birthweight-Hadlock III; (ıv) Birthweight-Hadlock IV; (v) Birthweight-Hadlock V.

**
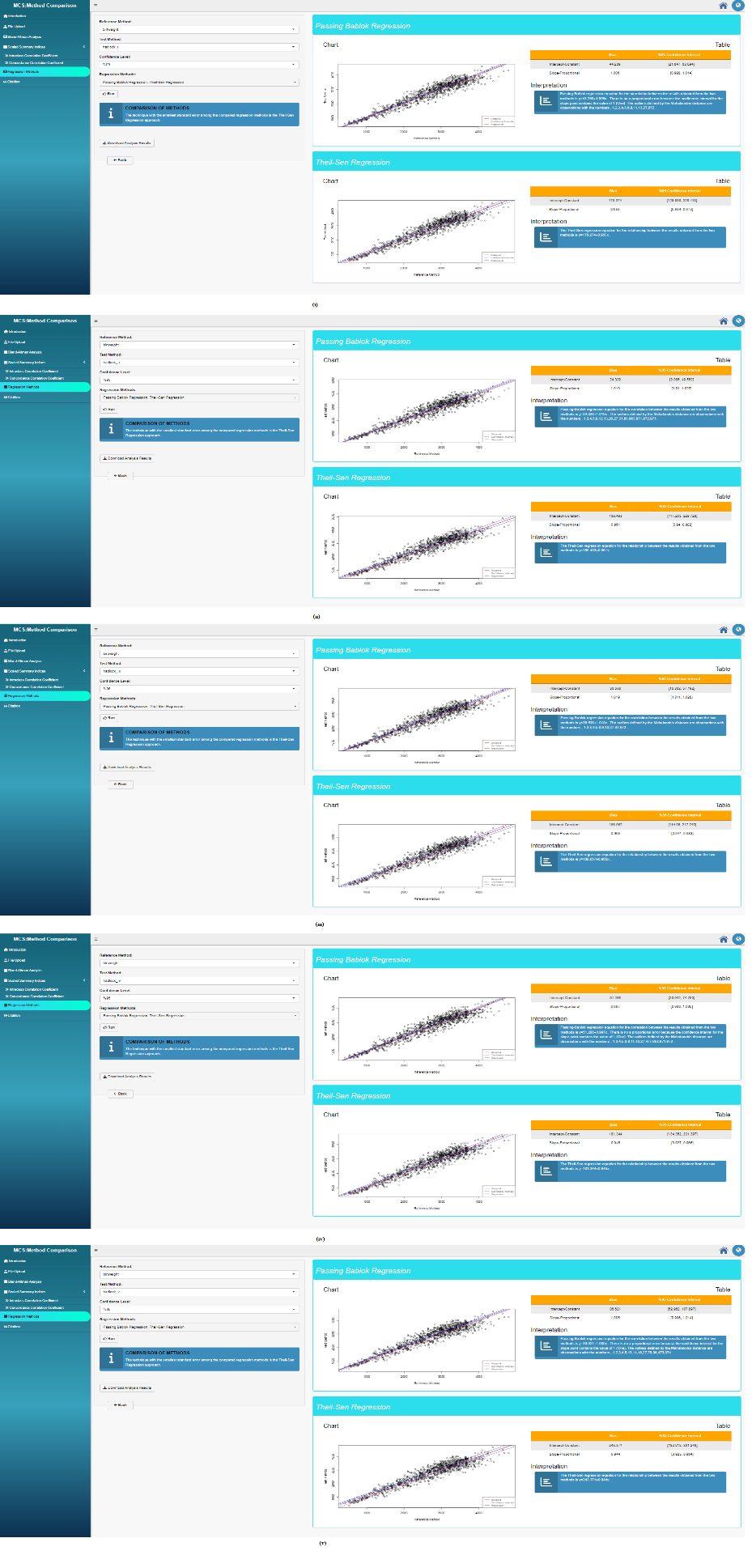
**

**Figure 5.** Screenshot of the "Regression Methods" module of the developed web-based software; (ı) Birthweight-Hadlock I; (ıı) Birthweight-Hadlock II; (ııı) Birthweight-Hadlock III; (ıv) Birthweight-Hadlock IV; (v) Birthweight-Hadlock V.
